# Introduction to the Greek Taxon Information System (GTIS) in LifeWatchGreece: the construction of the Preliminary Checklists of Species of Greece

**DOI:** 10.3897/BDJ.4.e7959

**Published:** 2016-11-01

**Authors:** Nicolas Bailly, Vasilis Gerovasileiou, Christos Arvanitidis, Anastasios Legakis

**Affiliations:** ‡ Institute of Marine Biology, Biotechnology and Aquaculture, Hellenic Centre for Marine Research, Heraklion, Crete, Greece; § Department of Biology, Section of Zoology and Marine Biology, University of Athens, Athens, Greece

## Abstract

The Greek Taxon Information System is an initiative of the LifeWatchGreece Research Infrastructure (ESFRI) that is resuming efforts to compile a complete checklist of all species reported from the Greek territory. Such an effort is necessary as a requirement for all signatories of the Convention on Biological Diversity (Greece is a signatory since 1994). Over an estimation published in 2004 according to which 50,000 species are present in Greece, belonging to most kingdoms except bacteria and viruses, a list of 35,000 valid species (and subspecies) has been assembled from previous national and European initiatives and specialized databases on various groups. A new database will be progressively set up in the LifeWatchGreece Infrastructure within the near future. Before the dissemination of this dataset, it is important that the checklists will be validated by specialists for each taxonomic group. The first step already accomplished was to build and publish Preliminary Checklists for some taxonomic groups of marine fauna, which have been validated by specialists on the basis of their expertise and secondary literature. The publication of these Preliminary Checklists is expected to increase the visibility and usability of the database in the future not only to the scientific community but also to the broader domain of biodiversity management, especially in cases where no such checklists have been published yet. The guidelines used to test the first taxonomic groups are presented in this paper.

### Keywords

Biodiversity, global species databases, biodiversity management, data management

## Introduction

LifeWatchGreece (LWG) aims at building a research e-infrastructure for biodiversity data (for genes, species, and ecosystems) including data from observatories, which is composed by a suite of electronic services (e-Services) and virtual laboratories (vLabs). Data will cover the terrestrial and freshwater life zones of Greece, while data for the marine life zone will cover the entire Mediterranean Sea. A portal is providing access to data and to the e-Services and vLabs. Like in all biodiversity information systems, a taxonomic backbone is essential (Bisby and Roskov 2010, Cochrane and Galperin 2009, Berendsohn et al. 2011, Martellos and Attorre 2012, Patterson et al. 2014). In particular, the complete list of all species occurring in Greece is necessary for a sustainable management of biodiversity of the country, balancing both exploitation and conservation. Such an effort is necessary as a requirement for all signatories of the Convention on Biological Diversity, including Greece since 1994. LifeWatchGreece is currently being developed in the context of the European ESFRI project LifeWatch (Basset and Los 2012).

Following the concrete efforts to compile checklists of living species in Greece per taxonomic group initiated in early 80s (Legakis 1979, Legakis 1989, Legakis 1998, Legakis 2005), the first attempt to provide a list of all the terrestrial and freshwater animal species hitherto reported from Greece was made in the context of the project GREFAUN (1998-2001). This project was carried out by the Zoological Museum of the University of Athens in collaboration with the Hellenic Zoological Society and funded by the General Secretariat of Research and Technology (GSRT). The project was set up in MS-Access and included approximately 23,000 taxa. With the addition of 3,500 marine species and using extrapolations, it was calculated that the total number of animal species reported from Greece would reach 40,000-50,000 species (Legakis 2004). Subsequently, the "Greek Biodiversity Database" project (2005-2008) was launched to provide the list of all species recorded in Greece by the Department of Zoology, School of Biology, at the Aristotle University of Thessaloniki (AUTH). The documented occurrence of species in the country was recorded in a database that was set up online in 2010 (Koukouras et al. 2010) from an AUTH server. The "Greek Biodiversity Database" had benefited from lists assembled per taxonomic group in the context of a series of PhD, MSc and BSc projects and from lists compiled under three European projects (ERMS, Costello et al. 2001; Fauna Europaea, Jong et al. 2014, Jong et al. 2015; Euro+Med Plantbase, Jury 2015) that were ultimately integrated under PESI (Jong et al. 2015). Although not completed, the database had not been updated since the project was terminated in 2010. Costello et al. (2014) explained the conditions for a successful long-term maintenance of biodiversity information systems that were established by short-term projects.

Within the LifeWatchGreece project, we have established the Greek Taxon Information System (GTIS) led by A. Legakis, as the Editor-in-Chief. Although GTIS does not form a formal follow-up of the previous projects, as it uses advanced methodologies and procedures, the data content is primarily based on them. A new database in PostGreSQL will be set up as an application in the LifeWatchGreece Portal, and some parts of the content will be also disseminated as a Scratchpad. The "Greek Biodiversity Database" did not meet these conditions of sustainability (Costello et al. 2014) on the contrary of the GTIS that is hosted in a long-term European infrastructure. The goals, activities and final data structure and tools will be detailed in a further publication.

On the way to build the GTIS, the first step is to publish Preliminary Checklists: they are primarily validated by specialist taxonomists on the basis of their current knowledge and secondary literature (synthetic works such as faunas, floras, previous checklists, etc.). The next step will be to elaborate Annotated Checklists based on primary literature including all comments about the taxonomy and the status of occurrence in Greece.

This paper presents the main guidelines and workflow for the production of the Preliminary Checklists. They have been currently tested on some marine taxonomic groups (i.e. Ascidiacea, Brachiopoda, Cephalopoda, Chaetognatha, Cumacea, Lophogastrida, Mysida, and Porifera) and will be applied for the future lists in the taxonomic scope of the GTIS (basically, all groups of biota). A number of Preliminary Checklists will be published within the present LifeWatchGreece online collection of papers in *Biodiversity Data Journal*. Specific details will be added in each checklist paper by taxonomic group. Some groups are already covered by recent annotated checklists (e.g. fish, flowering plants, and cetaceans) and will not be the subject of a Preliminary Checklist, but they will be included in the database with the proper bibliographic reference. This work also suggests that this type of publications is still needed to support the correct citations of databases.

## Material and methods

This section explains the general principles used for elaborating the Preliminary Checklists. Specific details for each taxonomic group will be given in the respective dedicated publications.

### Search for citations of the Greek Biodiversity Database

In order to justify the necessity for the publication of the Preliminary Checklists, we checked the citation frequency of the "Greek Biodiversity Database". We performed an advanced search for previous citations of the "Greek Biodiversity Database" in Google scholar, by searching for relevant phrases ("Greek Biodiversity Database / website / project / information system", "Checklist of marine species from Greece" for marine taxa) and for the URL anywhere in the articles. Surprisingly, just one citation of the "Greek Biodiversity Database" was found using the search engines only. Additionally, 13 citations were spotted for marine species under the reference Koukouras (2010) that was specially created by WoRMS (Vandepitte et al. 2015) for the list provided by the "Greek Biodiversity Database" during the European project PESI; however, 6 of them were from Encyclopedia of Life through the use of WoRMS data.

In total, only 7 scientific publications cited the Greek Biodiversity Database since 2010 indicating that stable paper-published references are still needed for crediting information systems in addition to the website itself.

### Delimitation of the geographic area

**Figure 1.**
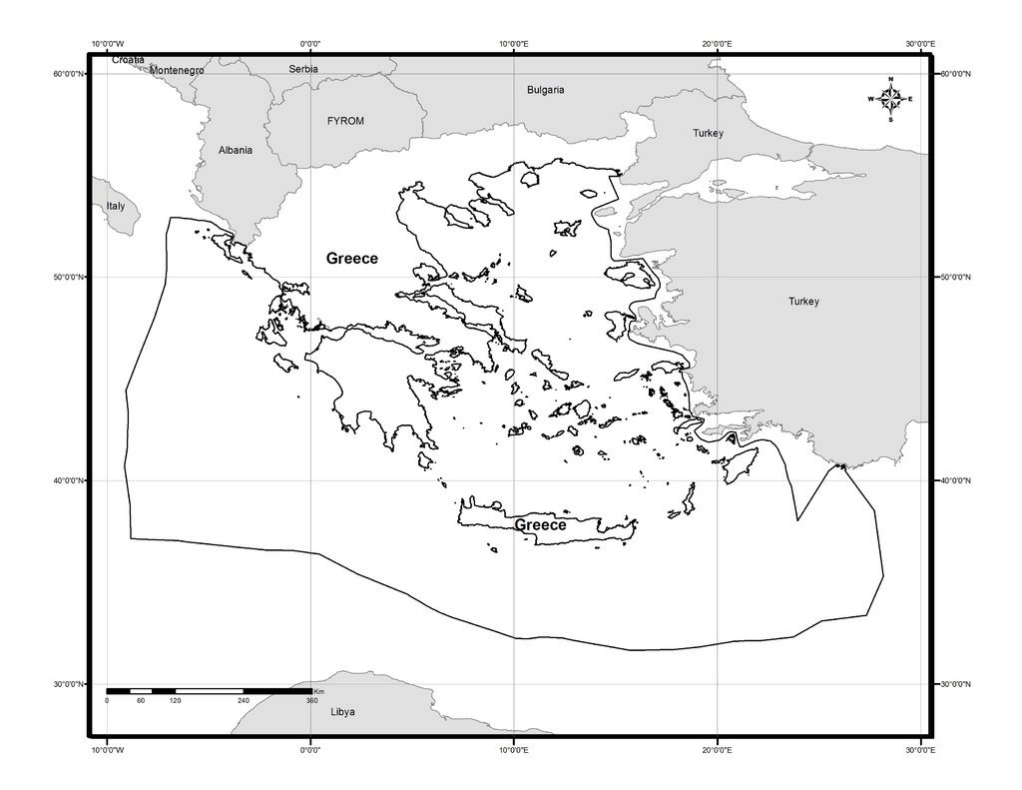
Map of the terrestrial and marine territories of Greece.

The geographic area covered (Fig. 1) is composed of the Greek territorial land, including freshwater bodies and the adjacent seas within the Greek "Exclusive Economic Zone (EEZ)" as delimited in the Marine Regions database (Claus et al. 2015).

### Higher hierarchy (classification)

The Preliminary Checklists were plugged into the classification of Ruggiero et al. (2015) as the main taxonomic backbone. Adaptations could be made by specialists below the order rank.

### Principles for the creation and validation of Preliminary Checklists

Creation of the list

The classification and species records for each taxonomic group, that were listed as present in Greece, were extracted from the following datasets: Greek Biodiversity Database; PESI; WoRMS/ERMS for Marine species; Fauna Europaea for terrestrial and freshwater animals, with E+MP for higher plants; relevant Global Species Databases such as AlgaeBase (Guiry and Guiry 2016) for Algae, FishBase (Froese and Pauly 2016) and Catalogue of Fishes (Eschmeyer and Fricke 2016) for fishes; dedicated Greek databases such as www.herpetofauna.gr for reptiles, the list of birds of Greece from the Hellenic Ornithological Society, and ELNAIS (Zenetos et al. 2015) for non-indigenous species. In some cases, species that were flagged as present in Europe or the Mediterranean Sea but not in Greek land/waters were also kept in the list for further check by specialists, especially for those groups that were not previously validated.

Extraction and conversion of datasets from previous databases to a new database structure

The species and name lists were firstly stored in a relational database used for construction and validation. A scratchpad entitled "Species List of Greece (SpeLoG)" was secondarily used for further dissemination, and will be integrated subsequently in the LifeWatchGreece Portal. The Greek Biodiversity Database was managed under MySQL as the back-end of the Content Management System Drupal. The new database is currently managed locally under MS-Access and will be eventually moved to the LifeWatchGreece infrastructure under PostGreSQL. The technical details of the database structure will be detailed in a further paper.

The followed classification below order rank was checked against reference databases such as Catalogue of Life, WoRMS and IPNI for plants according to the group. Current taxonomic status and current accepted names were also checked against PESI and its 3 components. In cases where different classification systems were followed by different databases, a rapid literature search was conducted for the latest phylogenies/classifications. Global Species Databases dedicated to one taxonomic group were also accessed when they were published online (e.g., Amphibia of the World, AmphibiaWeb, Chaetognatha of the World, etc.). As far as possible, feed-backs were sent to the managers of those databases when issues were raised during the comparisons.

Taxon names and their authority were matched against and corrected from the reference databases. Non-accepted names were kept in our internal database when they were used to report a species in Greece in a scientific publication but were not listed in the Preliminary Checklists.

References reporting species presence since 2000 were also researched (previous available checklists and new records). These were published in (a) the reference database for animals assembled by the Zoological Museum of the University of Athens and the Hellenic Zoological Society (Legakis 2015), (b) Greek databases (see Introduction), (c) a list of references assembled for PESI; and (d) a list currently compiled under LifeWatchGreece locating occurrence data and datasets in literature references. Finally, a more detailed search was conducted in several search engines (i.e. Google Scholar, Web of Science, Scopus, Mendeley, and Zotero), using keywords and tags such as the name of the group (scientific and common names in English and Greek) and the geographic area (Greece, marine regions, state divisions, islands, etc.), in order to find the most recent publications about the classification/phylogeny of the group.

Occurrences from Greek geographic areas were also searched in Global Species Databases which provide distribution data by country. However, point data with geocoordinates were not reported in this Preliminary Checklist step.

Validation

Individual checklists were exported from MS-Access as worksheets in MS-Excel files: one with the species list itself, one with the main relevant and potentially useful references. The files were sent to specialist taxonomists for a thorough examination of the lists for possible errors and omissions, with a request for relevant secondary literature. Several rounds of "listing – validation – corrections/additions" were sometimes needed until Preliminary Checklists were considered validated. For most taxonomic groups, we consulted Greek colleagues with a particular expertise in the fauna or flora of Greece, even if they were not taxonomists *sensu stricto.* In cases of lack of a national expert we invited international taxonomists to examine the lists in collaboration with Greek colleagues who could follow up and continue their updating in the future. Specialists who validated the checklists will be main authors of the respective published lists and cited as taxonomic editors for every record in the database as well.

Vocabulary

*Status of occurrence in the country*

The following terms are used in the database and the Preliminary Checklists for the status of occurrence of species:

- Present (including non-indigenous species recorded in Greece);
- Possible: this term can be used for doubtful reports (e.g., about the identification or location), or for species present in Europe/Mediterranean but not reported in Greece so far;
- Absent for mistaken records and misidentifications included in previous databases or publications. All species wrongly reported from Greece will be stored in the database as mistakes so that such errors will not be disseminated anymore.

*Reported - recorded*

Throughout the initiative, we will use the following terms consistently:

- Reported: a species observed or collected in the country’s territory, whatever the validity of that report.
- Recorded: the report of the species has been positively confirmed. The set of recorded species represents the fauna and flora of the country that is currently known to occur in Greece. The "Recorded" status of a species can be subsequently changed back to "Reported" when a new doubt about the presence of the species is raised.

To summarize: {Recorded} = {Reported} - {Possible, Absent}. The status Present is then equal to the status Recorded. The two terms are used in different context: the vocabulary {Reported, Recorded} makes reference to an action of observation, while the {Present, Possible, Absence} lists the statuses of occurrence in the country.

## Discussion

The Greek Taxon Information System in LifeWatchGreece is an initiative with a complementary role to previous individual projects on biodiversity databasing which had never been integrated or systematically updated at least within an organized national framework. A notable example is the Greek Biodiversity Database and its website that had not been widely known and used given the surprisingly low number of citations for such an initiative. This was also apparent during the checklist validation process as most of the contacted specialists were not aware of the existence of this database although the majority of collaborators came from Greece. We expect that the publication of taxonomic papers (e.g. Preliminary Checklists) in scientific journals will increase the visibility and usability of such type of biodiversity data, not only to the scientific community but also to the broader domain of biodiversity management, especially in cases when checklists had not been previously published. Online databases, like scratchpads (e.g. SpeLoG application within LifeWatchGreece) provide the ability for users to download species lists, match name lists from their own work, or report errors and additions. Although online databases make data accessible to a wide range of end-users, they are still problematic in terms of referencing, because electronic data are volatile compared to data listed in scientific publications. So, readers should be encouraged to check active online databases for their updated content.

The procedure followed within the GTIS initiative revealed taxonomic groups under different status of knowledge in Greece: (a) well-covered (e.g. Lepidoptera, Pisces, Mammalia); (b) not recently updated (e.g. Porifera, Sipuncula, Ascidiacea); (c) never listed before although sporadic records have been published in scattered literature sources (e.g. Chaetognatha); and (d) never studied (e.g. Ctenophora, Tardigrada), due to the taxonomic impediment (= regional lack of specialists). All groups of biota will be progressively listed and published under GTIS. Preliminary lists have been already tested for some marine taxonomic groups, such as Ascidiacea, Brachiopoda, Cephalopoda, Chaetognatha, Cumacea, Lophogastrida, Mysida, and Porifera. Preliminary Checklists will not be compiled for well-covered groups with checklists recently published such as Pisces (Papaconstantinou 2015, Barbieri et al. 2015), Lepidoptera (Gozmány 2012), flowering plants (Dimopoulos et al. 2013), or known to be in preparation, for instance volumes of *Fauna Graeciae* collection and publications of the Hellenic Botanical Society. However, these checklists will be also directly integrated in GTIS.

The following challenges were encountered during the compilation of the Preliminary Checklists: (a) checklists compiled from individual sources required thorough taxonomic updates; (b) Global Species Databases do not necessarily report species by country; (c) specialists are lacking in Greece for a number of groups; (d) voluntary scientific work is challenging. The publication of checklists and data papers could be an immediate reward to collaborators who validate the Preliminary Checklists: a publication still constitutes a better credit than just citing their name in the database/scratchpads records to which it will be associated in all cases.

A gap analysis for taxonomic groups that could not be validated, combined with the collaboration of local and international scientists, could stimulate future research on understudied taxa. A strategic plan should be developed to fill the gaps including the involvement of research/academic authorities, scientific societies and citizen science initiatives for completing the study of the taxonomy for all species present in Greece. Local experts do not need to be taxonomist specialists of a given group, but may be good experts of the local fauna and/or flora (which implies they have a good knowledge of taxonomy in general), and serve as national experts and focal points for given groups; they could then ensure the monitoring of the knowledge for these groups in Greece. For this reason, the LifeWatchGreece Infrastructure involves a wide network of research and academic institutions all over Greece. The overall GTIS initiative is open to collaboration with taxonomists from the Greek, European and World scientific community who are interested in contributing to this effort.
